# Intracranial Drain-Related Intracerebral Hemorrhage in Two Sporadic Cerebral Amyloid Angiopathy Patients

**DOI:** 10.3233/ADR-240086

**Published:** 2024-07-23

**Authors:** Dimitri Renard, Birama Sangare, Ansma Youssouf, Eric Thouvenot

**Affiliations:** a Department of Neurology, CHU Nîmes, University Montpellier, Montpellier, France; b Institut de Génomique Fonctionnelle, CNRS UMR5203, INSERM 1191, University Montpellier, Montpellier, France

**Keywords:** Alzheimer’s disease, cerebral amyloid angiopathy, decompressive craniectomy, intracranial drain, intracerebral hemorrhage

## Abstract

Alzheimer’s disease and cerebral amyloid angiopathy (CAA) are often associated. Amyloid accumulation within leptomeningeal and small/median-sized cerebral blood vessels in CAA results in vessel fragility, leading to spontaneous leptomeningeal bleeding, lobar intracerebral hemorrhage (ICH) and cerebral microbleeds. CAA is also associated with non-traumatic subdural hematoma. The role of CAA-related vessel fragility in hemorrhagic complications after trauma, brain surgery, and intracranial drain insertion in CAA is unknown. We present two sporadic CAA patients with intracranial drain-related ICH, probably due to different underlying mechanisms, related to indirect and direct CAA-associated vessel fragility.

We present two sporadic cerebral amyloid angiopathy (CAA) patients with intracranial drain-related intracerebral hemorrhage (ICH). Informed consent was obtained from all individual participants included in the study.

The first patient (Fig. 1) is a 71-year-old man with an 8-year history of sporadic CAA (*APOE* E2/E4 genotype, with positive cerebrospinal fluid (CSF) CAA biomarkers and amyloid PET) who presented with traumatic extra- and subdural hemorrhage after major blunt trauma without fracture. After two days, the patient’s state worsened and increase of hemorrhage was observed on CT. The extra- and subdural hemorrhages were evacuated, and a subdural drain was inserted. Postoperative CT showed multiple lobar and deep (i.e., thalamic) ICH in the brain regions earlier compressed by the initial extra/subdural hemorrhage located in front of the subdural drain, together with intraventricular hemorrhage.

**Fig. 1:**
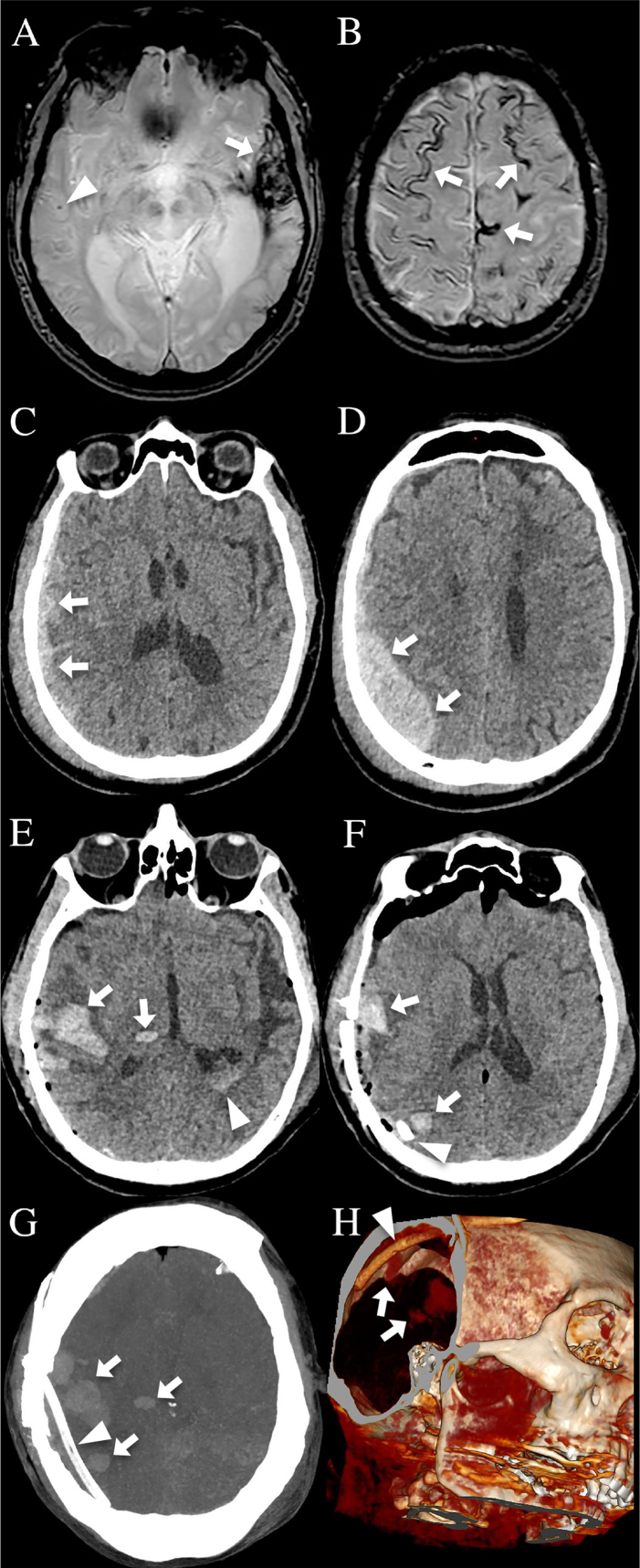
CAA patient 1 showing chronic ICH (A, arrow), cerebral microbleed (A, arrowhead), and cortical superficial siderosis (B, arrows) on MRI performed six months before head trauma. After major blunt head trauma, sub- (C) and extradural (D) hemorrhage can be observed on CT. After hemorrhage evacuation, a subdural drain (F, G, and H, arrowhead) was inserted complicated by multiple lobar and deep ICH intracerebral (E-H, arrows) in brain regions earlier compressed by the initial extra/subdural hemorrhage and in front of the subdural drain (E and F are standard CT images, G 2D and H 3D multiplanar reconstruction CT images) together with intraventricular hemorrhage (E, arrowhead) on postoperative CT.

The second patient (Fig. 2) is a 72-year-old man with a 12-year history of sporadic CAA (*APOE* E2/E3 genotype, with positive CSF CAA biomarkers and amyloid PET) presenting with right frontal lobar ICH recurrence associated with intraventricular hemorrhage extension. After three days, the patient’s state worsened, and CT showed hydrocephalus probable related to intraventricular hemorrhage. A left frontal intraventricular drain was inserted. Postoperative CT showed ICH surrounding the intraventricular drain.

**Fig. 2:**
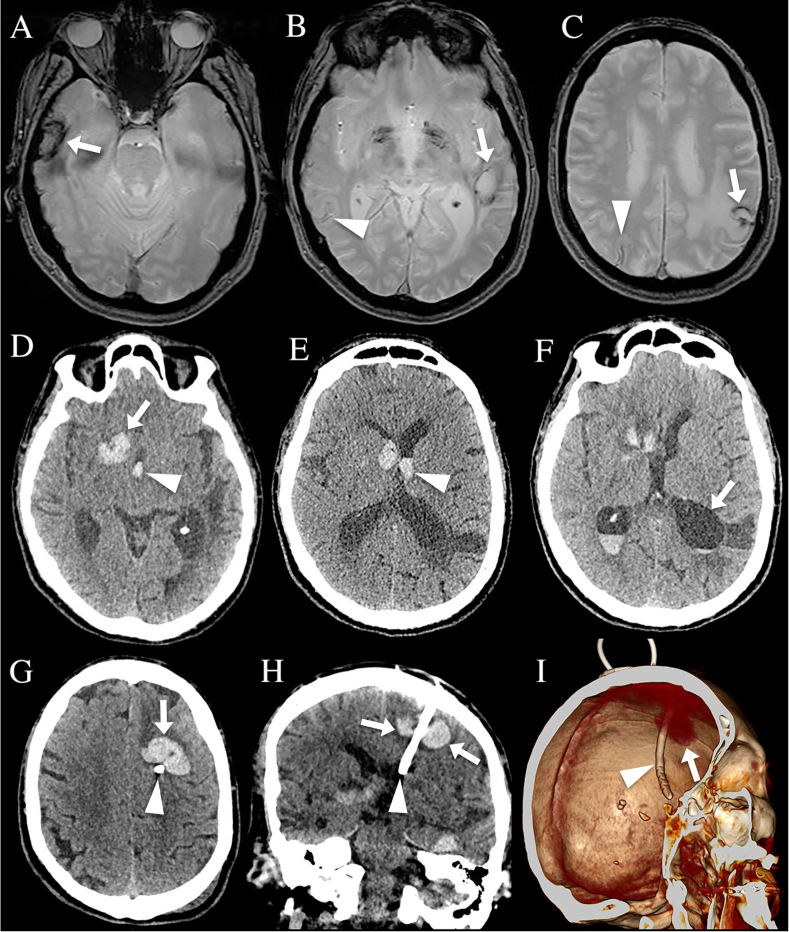
CAA patient 2 showing multiple chronic ICH (A-C, arrows) and cortical superficial siderosis (B and C, arrowheads) on MRI, four months before recurrence of acute ICH. Acute right frontal lobar subcortical ICH (D, arrow) associated with intraventricular hemorrhage extension (D and E, arrowheads) can be observed on CT. After three days, the patient’s state worsened and CT showed occurrence of hydrocephalus (F). A left frontal intraventricular drain was inserted. Postoperative CT showed ICH (arrows) surrounding the intraventricular drain (arrowhead) an axial (G) and coronal (H) CT images and on 3D multiplanar reconstruction images (I).

Amyloid accumulation within leptomeningeal and small/median-sized cerebral blood vessels in CAA results in vessel fragility, leading to spontaneous leptomeningeal bleeding, lobar ICH, and cerebral microbleeds. CAA is also associated with non-traumatic subdural hematoma.^1,2^ The role of CAA-related vessel fragility in hemorrhagic complications after head trauma, brain surgery, and intracranial drain insertion in CAA is unknown.

CAA diagnosis in both presented patients was essentially based on MRI features fulfilling the CAA diagnostic criteria. Although not included in the diagnostic criteria (since CSF and PET changes are not specific since they can be observed also in Alzheimer’s disease for instance, associated or not with CAA), CSF biomarker and amyloid PET abnormalities can be indicative for CAA diagnosis. In the first patient, the association of lobar and deep ICH and intraventricular hemorrhage in front of the subdural drain evokes a pressure-related mechanism (i.e., compression followed by decompression, intracranial hypertension followed by drain-related hypotension) rather than direct parenchymal traumatic drain-induced injury. Intracranial hypotension after spinal or cranial surgery is thought to be related to intraoperative CSF leak resulting in stretching and occlusion of cerebral veins resulting in hemorrhagic venous infarction. The most frequent form is these kinds of hemorrhages is remote cerebellar hemorrhage (with the most frequent bleeding pattern the so-called “zebra sign”, named after the streaky bleeding pattern from subarachnoid hemorrhage distributed over the superior surface of the cerebellum) as a complication of spinal or supratentorial surgery, whereas also ICH and intraventricular hemorrhage or a combination of these can be encountered.^3^ We hypothesize that the surgery- and drain-related decompression after brain compression of brain parenchyma with underlying arterial and venous vessel fragility due to CAA may have caused the post-surgery/drain ICH and intraventricular hemorrhage.^4,5^

In the second patient, direct brain injury associated with the insertion of the intraventricular hemorrhage can be suspected because of the co-localization of the ICH around the drain.

During the 1990 s, reports based on small patient numbers did not suggest higher surgical bleeding risks in CAA patients.^6–8^ However, in 2023 Yanagawa et al., reporting 79 patients with surgical removal of lobar ICH, showed that CAA histology was associated with postoperative rebleeding (especially in patients receiving prior anticoagulation therapy) compared with non-CAA patients.^9^ To the best of our knowledge, these are no reported data on CAA patients treated with intracranial drain only (like in patient 2 reported here).

Decompressive craniectomy and intracranial drains for traumatic and non-traumatic ICH, extra/subdural and intraventricular hemorrhage are often performed only based on CT imaging. In order to study the potential bleeding complications after decompressive craniectomy and intracranial drain insertion in CAA compared with non-CAA patients, pre-surgical MRI should be performed in order to detect CAA-related MRI features. These data might help clinicians and surgeons in guiding the management of CAA patients needing decompressive craniectomy and/or intracranial drains.

In CAA, the presence of an *APOE4* and *APOE2* allele is associated with higher risk of ICH recurrence. Further studies must be performed in CAA patients undergoing decompressive craniectomy or with inserted intracranial drains to assess if *APOE* genotype also influences specifically surgery-related bleeding complication.

## AUTHOR CONTRIBUTIONS

Dimitri Renard (Conceptualization; Data curation; Formal analysis; Investigation; Methodology; Project administration; Resources; Supervision; Validation; Visualization; Writing – original draft; Writing – review & editing); Birama Sangare (Conceptualization; Methodology); Ansma Youssouf (Conceptualization; Methodology); Eric Thouvenot (Supervision).

## Data Availability

Data can be obtained on reasonable request.
